# A novel *SPEG* mutation causes non-compaction cardiomyopathy and neuropathy in a floppy infant with centronuclear myopathy

**DOI:** 10.1186/s40478-018-0589-y

**Published:** 2018-08-29

**Authors:** Haicui Wang, Anne Schänzer, Birgit Kampschulte, Hülya-Sevcan Daimagüler, Thushiha Logeswaran, Hannah Schlierbach, Jutta Petzinger, Harald Ehrhardt, Andreas Hahn, Sebahattin Cirak

**Affiliations:** 1 Department of Pediatrics, University Hospital Cologne, Kerpener Straße 62, 50937 Cologne, Germany; 2 Center for Molecular Medicine Cologne (CMMC), University of Cologne, Robert-Koch-Str. 21, 50931 Cologne, Germany; 3 Institute of Neuropathology, Justus Liebig University Giessen, Arndtsr.16, 35392 Giessen, Germany; 4 Department of General Pediatrics and Neonatology, Center of Child and Youth Medicine, Justus Liebig University, Giessen, Germany; 5 Department of Pediatric Cardiology, Center of Child and Adolescent Medicine, Justus Liebig University, Giessen, Germany; 6 Department of Child Neurology, Center of Child and Adolescent Medicine, Justus Liebig University Giessen, 35392 Giessen, Germany

Bi-allelic mutations of the striated muscle preferentially expressed protein kinase (*SPEG,* OMIM 615959) were demonstrated to cause centronuclear myopathy (CNM) [1, 19]. SPEG interacts with myotubularin 1 (MTM1) in myofibers and mechanism of disease was indicated as the mishandling of Ca^2+^ in the sarcoplasmic reticulum (SR) of skeletal muscle and cardiac muscle [6, 14].

Here we report a peculiar CNM case caused by a novel homozygous *SPEG* stop mutation c.7119 C > A (p.Y2373*) (Additional file 1: Figure S1 and S2) resulting a C terminal truncation with loss of the last three important domain Ig-like/ Fibronectin type III/ Protein kinase domains, including the myotubularin interaction region (amino acid 2530–2674 mouse Speg) in both highly expressed SPEG isoforms in skeletal and cardiac muscles (SPEGα and SPEGβ). The male patient was the second child of consanguineous healthy Turkish parents. He was born spontaneously after an uneventful pregnancy at term and required intubation for ventilator support immediately after birth. The newborn presented as a severe floppy infant without spontaneous movements, he had a contracture of the right ankle and lack of deep tendon reflexes. After a few week he gained antigravity strength and was able to raise hands and feet, however, still was depending on gastric tube feeding due to swallowing difficulties. Assisted ventilation could be weaned off at 10 weeks of age despite hypercapnia.

In addition, echocardiography at age 10 weeks revealed a shortening fraction of 30% and a normal inner diameter of the left ventricle, but disclosed enlarged atria, and abnormal trabeculation of the left ventricle and intra-trabecular recesses as pathognomonic of left-ventricular non-compaction (LVNC) (Fig. 1l-m and Additional file 2: video sequence 1 and 2). He was discharged home to palliative care at 13 weeks of age. The parents denied further intubation and transport to the intensive care unit, and the boy deceased at 19 weeks of age due to an upper respiratory tract infection.

**Fig. 1:**
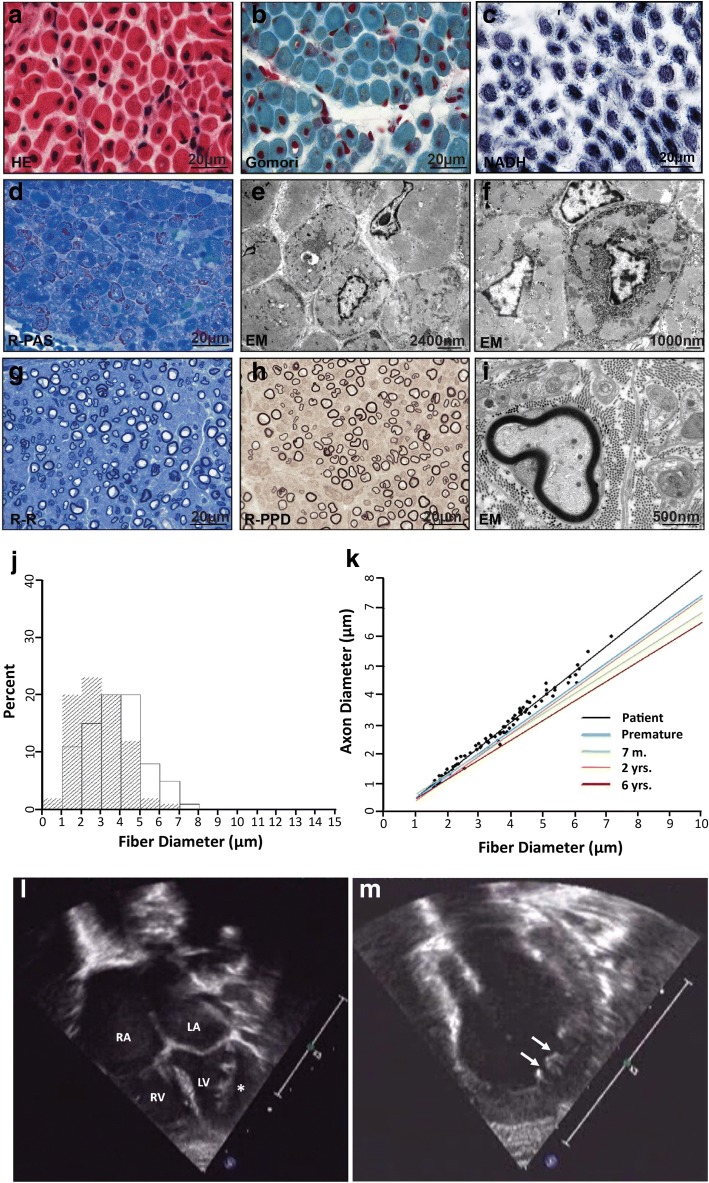
**a**-**i** Muscle biopsy performed at age of eight weeks shows a myopathy with increased variation in muscle diameter and internalized nuclei at H&E (**a**) and Gomori trichrome (**b**) stained cryosections consistent with a centronuclear myopathy. In **c** NADH staining the muscle fibers show disturbance of the internal myofiber architecture with central dark staining and pale surrounding halo. Many fibers show increased glycogen in PAS-stained semithin sections (**d**) and at ultrastructural analyses with disturbance of the myofiber architecture (**e**, **f**). In the sural nerve biopsy, the number of myelinated fibers appears slightly decreased (**g**) and the axons have mostly thin myeline sheats (**h**, **i**). **j**-**k** Morphometric analyses of the sural nerve biopsy at age eight weeks. **j** The histogram shows the frequency of distribution of axonal (hatched) and fiber diameters. The diameter distribution is unimodal with a diameter between 1 and 8 μm and shows an increased frequency of small fibers and axons for this age. **k** Analyzing the correlation of fiber to axonal diameter the patient has thinner myelin sheaths (slope = 0.87) compared to normal controls in literature [3, 7] (slope = 0.77) (m = month, yrs. =years). **l**-**m** Echocardiography of the patient at age 10 weeks. Four-chamber view (**l**) and isolated view on the left ventricle (**m**) demonstrating enlarged atria as suggestive of restrictive cardiac dysfunction, and abnormal trabeculation and intra-trabecular recesses (arrows and asterisk) as characteristic of left-ventricular non-compaction cardiomyopathy

Creatine kinase (CK) values were normal. At 3 weeks of age, electroneurography of the right median nerve displayed a motor nerve conduction velocity (NCV) of 20 m/s and amplitudes of 0.5 mV distally and 0.6 mV proximally, while the NCV of the left peroneal nerve was 23 m/s with a distal amplitude of 0.7 and a proximal amplitude of 0.5 mV. The norm values are from our in-house data based on 25 subjects ranging in age from 0 to 3 months, with in-house norm values (CV) of 25.4 ± 2.3 m/s for the median and 24.6 ± 2.0 m/s for the peroneal nerve. The in-house CVs for the amplitudes are 3.3 ± 0.35 mV for the median and 3.2 ± 0.4 mV for the peroneal nerve. We regard the values below twice the standard deviation as abnormal. Our norm values fit best with those obtained in the previous study by Parano et al. [13], who investigated 20 children ranging in age from 0 to 1 months and 23 ranging in age from 1 to 6 months with CV 25.43 ± 3.84 for the median and 22.43 ± 1.22 m/s for the peroneal nerve. The CVs for the amplitudes were 3.0 ± 0.31 mV for the median and were 3.06 ± 1.26 mV for the peroneal nerve according to Parano et al. [13]. These norm values also fit the descriptions in the book “Pediatric Clinical Electromyography” [8]. In our patient, NCVs were within normal limits but the amplitudes of both, the median and the peroneal nerve, were substantially below the normal range (> SD) when compared to in-house data derived from 25 children ranging in age from 0 to 3 months, and when related to normal values determined by Parano et al. [13], who investigated 20 newborns within the first month of life. In summary, our findings were well compatible with an axonal neuropathy.

Diagnosing a milder form of neuropathy in newborns and younger infants is challenging due to rapid maturation of the peripheral nervous system in conjunction with substantial individual variability, and limited normative histological and electrophysiological data. Peripheral nerve myelination begins at approximately the 15th week of gestation and ends 3 to 5 years after birth. Normal values for nerve conduction velocities (NCVs) and compound muscle action potentials (CMAPs) in newborns, infants, and young children have been determined by several authors [8, 13], and found that the NCV is approximately half of the adult values at birth and increase differently in various nerves. Similarly, CMAPS triple in amplitude for the median nerve and double in size for the peroneal nerve as the child matures. No sensory nerve action potentials could be elicited with surface electrodes by retrograde stimulations of the right median and the left sural nerve. Electromyography of the left deltoid muscle displayed polyphasic motor unit potentials of small amplitude and short duration, consistent with a congenital myopathy. We decided to perform a combined nerve and muscle biopsy in order to investigate the morphological pathology leading to these electrophysiological findings of an axonal neuropathy and of myopathic changes in the electromyograph.

A combined biopsy of the right sural nerve plus the right lateral vastus muscle was performed at the age of 2 months (further technical processing details in the supplementary material). The muscle biopsy showed typical histological findings of a centronuclear myopathy with increased variation of fiber diameter with central localization of nuclei in most of the fibers (Fig. 1a, b) and in the NADH staining (Fig. 1c) central dark staining with pale surrounding halo highlighting the disturbance of the myofibrillar architecture. Antibodies against MHC-slow (Type I fibers, Additional file 1: Figure S3d) displayed no fiber disproportion. Many fibers showed a disturbed myofiber architecture with abundant glycogen in PAS-stained semithin sections (R-PAS) and at the ultrastructural level, (Fig. 1d, e, and f). Staining with developmental and fetal myosin and vimentin showed upregulation in many muscle fibers and as a hallmark of genetically confirmed myopathies, compatible with an increased regeneration as published earlier [11, 15] (Additional file 1: Figure S4). The patient was born at term and the muscle biopsy taken at the age of 2 months. At this age usually, there is no developmental myosin expression at all and even the fetal myosin expression shall be almost switched off [2, 16]. In contrast, we observe in our patient’s biopsy in almost every second fiber fetal myosin, suggesting that SPEG may lead to increased muscle degeneration/regeneration or affect maturation of myofiber.

In the sural nerve biopsy, the myelinated fibers appeared slightly decreased (Fig. 1h). Most fibers showed thin myelin sheaths which were confirmed by morphometric analysis, but no Schwann cell proliferation or misfolding of myelin was observed (Fig. 1g, h, i, k). The fiber diameter distribution was age-related unimodal within 1–8 μm, reflecting an increased frequency of small fibers and axons for this age (Fig. 1g, h, j) [3, 7]. Normal morphometric values for sural nerve biopsies in small children are spares but collectively, these findings together with the reduced amplitudes of the median and peroneal nerves provide cumulative evidence that an axonal neuropathy may represent a further feature of patients with SPEG mutations.

Mutations in several genes associated with CNM have been described to cause Charcot-Marie-Tooth neuropathies. For example, MTM1 mutations are (OMIM 310400) causing CNM, while mutations in other myotubularin-related proteins (MTMR2, MTMR13) cause CMT4B1 (OMIM 601382) and CMT4B2 (OMIM 604563) featured with demyelination and myelin outfoldings of the nerve, probably due to the disruption of membrane homeostasis or vesicle traffic in Schwann cell myelination as MTM1 and MTMRs maintain the homeostasis of the membrane phosphoinositides [18]. In the case of dynamin 2 (*DNM2,* OMIM 160150, 606482), CMT causing mutations impaired myelination with clathrin-mediated endocytosis [17]. Since SPEG interacts with the phosphatase and coiled-coil domains of the MTM1 and is co-localized in alignment with the terminal cisternae of the SR [1], it might play an essential role in membrane homeostasis or vesicle traffic for myelination.

Non-compaction cardiomyopathy might be frequently overlooked as it is commonly misdiagnosed as dilated cardiomyopathy. Since so far, all but one CNMs caused by *SPEG* mutations have also been associated with dilated cardiomyopathy (DCM) [1, 19]. Mutations of *Speg* leads to a dilated cardiomyopathy in mice and immature myocytes have been observed in the hearts of *Speg* mutant mice [9], which might be due to the downstream mishandling of Ca^2+^ via regulation of the Junctional Membrane Complex (JMC) activity by Speg [14]. Among the Ca^2+^ handling genes, calsequestrin2 (*CASQ2*) mutations [5] and Ryanodine receptor 2 gene (*RYR2*) mutations were reported to be LVNC causing [12]. Mutations in Titin, downstream effector of Ca^2+^ handling proteins for E-C coupling also cause LVNC [4], due to impairment in protein folding, stability and binding to a key cardiac and skeletal muscle protein telethonin. As Ca^2+^ handling is crucial for heart development and function, further fine evaluation of different mutations on Ca^2+^ handling might lead to an explanation how SPEG mutations cause DCM or LVNC.

Taken together, here we describe a novel *SPEG* mutation c.7119 C > A (p.Y2373*) which causes CNM and expand SPEG-associated phenotypes to neuropathy and non-compaction cardiomyopathy, which indicates further overlapping phenotypes with other CNM disease-causing genes, such as Dynamin 2 and Titin. Moreover, it also suggests a common CNM disease mechanism in membrane homeostasis and trafficking. Together with the newly reported truncating mutation which caused congenital myopathy without centralized nuclei [10], the patient phenotype spectrum now expands to a new grade. However, the clinical spectrum of SPEG-associated phenotypes remain to be deciphered by future identification of additional patients. We hypothesize that different functional disruption of CNM related proteins can shift the genotype further to other manifestation. Thus, a further deep evaluation of protein isoform function such as membrane homeostasis, vesicle trafficking, Ca^2+^ handling, with differential impact of mutations may help to correlate the genotype-phenotype better and will provide the basis for new molecular and personalized treatment strategies [19].

## Additional files

Additional file 1: Supplementary data. (PDF 791 kb)

Additional file 2: Enclosed video sequence 1 and 2 showing a four-chamber view and an isolated view of the left ventricle that depict restrictive cardiac malfunction in left-ventricular non-compaction cardiomyopathy. (ZIP 908 kb)
