# Supplementary material for: A novel SPEG mutation causes non-compaction cardiomyopathy and neuropathy in a floppy infant with centronuclear myopathy
Source: Acta Neuropathol Commun. 2018 Aug 29;6:83. doi: 10.1186/s40478-018-0589-y (PMC6114030; doi:10.1186/s40478-018-0589-y)
Supplement: Supplementary file 1 — Supplementary data. (PDF 791 kb) [file 40478_2018_589_MOESM1_ESM.pdf]

Material and Methods

Genetic Analysis

We performed whole exome sequencing of the patient at three months of age after obtaining parental informed consent for our ethically approved study from the local institutional review board in Cologne/Germany. Genomic DNA of the index patient was extracted from blood with the QIAamp DNA blood mini kit (Qiagen, Germany) and underwent whole exome sequencing (WES) on an Illumina High Seq 4000 Sequencer (Illumina, USA), using the SureSelect Human All Exon V.6_r2 (Agilent, USA) enrichment kit and a paired-end 75 bp sequencing protocol according to the manufacturer's best-practice protocol. Data analysis was performed with the Varbank V.2.25 exome pipeline of the Cologne Centre for Genomics (https://varbank.ccg.uni-koeln.de/), including alignment against the human reference genome hg19 and variant calling [5]. Sequencing data were filtered for rare (minor allele frequency <0.1%), homozygous variants (allele read frequency of 75%–100%) in accordance with the expected autosomal recessive mode of inheritance with the consanguineous familiar background. The mean coverage was 85%, 10x coverage for 96,3% and 30x coverage for 89% of target sequences. Variants affecting protein sequence or splice sites were considered. We used the best-practice filtering scheme based on the American College of Medical Genetics and Genomics (ACMG) guidelines [5] and common prediction tools for variant interpretation (Mutation Taster (http://www.mutationtaster.org/), gnomAD (http://gnomad.broadinstitute.org/gene/), ClinVar (https://www.ncbi.nlm.nih.gov/clinvar/), GME (http://igm.ucsd.edu/gme/data-browser.php) and ExAc (http://exac.broadinstitute.org)). Dideoxy sequencing was performed for confirmation and cosegregation of variants (Supplementary Table 1 and Supplementary Figure 1).

WES revealed a homozygous stop mutation in *SPEG* c.7119 C>A in Exon 30 causing p.Y2373* according to NM_005876.4. The mutation was not reported in the Exome Variant Server, gnomAD, ClinVar, and 1000 genomes databases nor has not been published before elsewhere to the best of our knowledge. The variant was classified as pathogenic by the ACMG standards and criteria [5].

Muscle and nerve morphology

Unfixed skeletal tissue was snap frozen and 6 µm cryosections were stained with hematoxylin and eosin (HE), gomori trichrome (Gomori) and nicotinamide dinucleotide (NADH). Immunohistochemical analysis was performed on cryosections using a Bench Mark XT automatic staining platform (Ventana, Heidelberg, Germany) with the following primary antibody: anti NCL-MHCs (1:1000, Novocastra), anti NCL-MHCd (1:20, Novocastra), anti NCL-MHCn (1:20, Novocastra), anti-Vimentin (V9) (1:500, M0725 Dako). The sections were examined using a Nikon Eclipse 80i microscope equipped with a CCD camera.

Small skeletal tissue was fixed with 6% glutaraldehyde/0.4 M phosphate buffered saline (PBS) and the sural nerve tissue was fixed with 3.9% glutaraldehyde/0.4 M PBS [9] and were processed with a Leica EM TP tissue processor. From resin embedded tissue 1-2 µm sections were stained with methylene blue, 2% p-phenylenediamine (PPD), and Periodic acid-Schiff (PAS). For electron microscopy, ultrathin sections were contrasted with 3% lead citrate trihydrate with a Leica EM AC20 (Ultrastain kit I and II) and were examined using a Zeiss EM 109 transmission electron microscope equipped with a Slowscan-2K-CCD-digital camera (2K-wide-angle Sharp:eye).

Morphometric analyses of the sural nerve were performed at a Zeiss EM109 transmission electron microscope equipped with a CCD camera with ImageSp-program. The axonal and fiber diameter of 80 fibers were analyzed at magnification x

7000 and compared to published normal controls [3] [4]. Statistics were performed using the R software package.

Supplementary Figure 1 Patient Pedigree

Patient Pedigree shows the parents are heterozygotes carriers while the patient is a homozygous carrier in SPEG gene (NM_005876, c.7119 C>A, p.Y2373*). Sanger sequencing traces showed gDNA sequence corresponding to the mRNA position 7115 to 7123 (NM_005876).

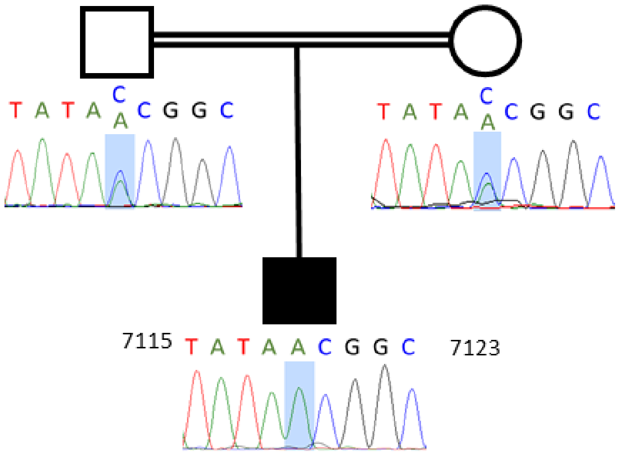

Supplementary Figure 2 Human SPEG protein domain organization

Human SPEG protein domain organization according to the description in NCBI and Uniprot, based on tissue-specific RNA expressions of GTEx database. Three putative isoforms might correspond to mouse SPEG isoforms SPEGα, SPEGβ, and APEG1, the first two isoforms are highly expressed in skeletal and cardiac muscle, while the APEG1 is the aortic specific isoform. The brain isoform sequence in human so far is uncertain [8].

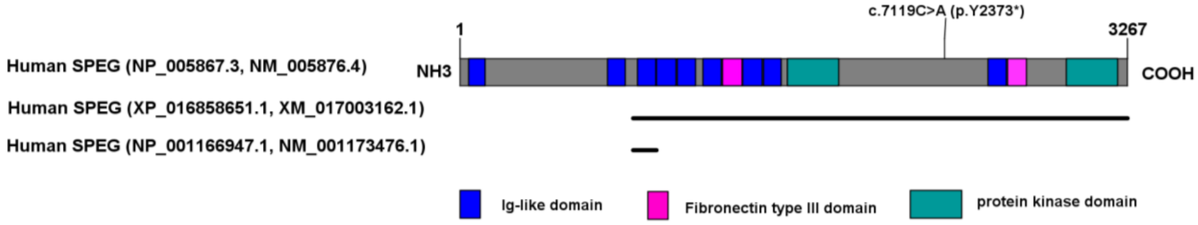

Supplementary Figure 3 Histopathological examination of patients' muscle biopsies at high magnification

Muscle biopsy performed at age of eight weeks at higher magnification shows typical histological findings of centronuclear myopathy a with marked variability in fiber size and numerous centrally placed nuclei surrounded by perinuclear halo at H&E (a) and Gomori trichrome (b) stained cryosections. In (c) NADH staining the muscle fibers show central dark staining with pale surrounding halo highlighting the disturbance of the intermyofibrillar architecture whereas a radial arrangement is not obvious. (d) Antibodies against MHC-slow (Type I fibers) show no fiber disproportion.

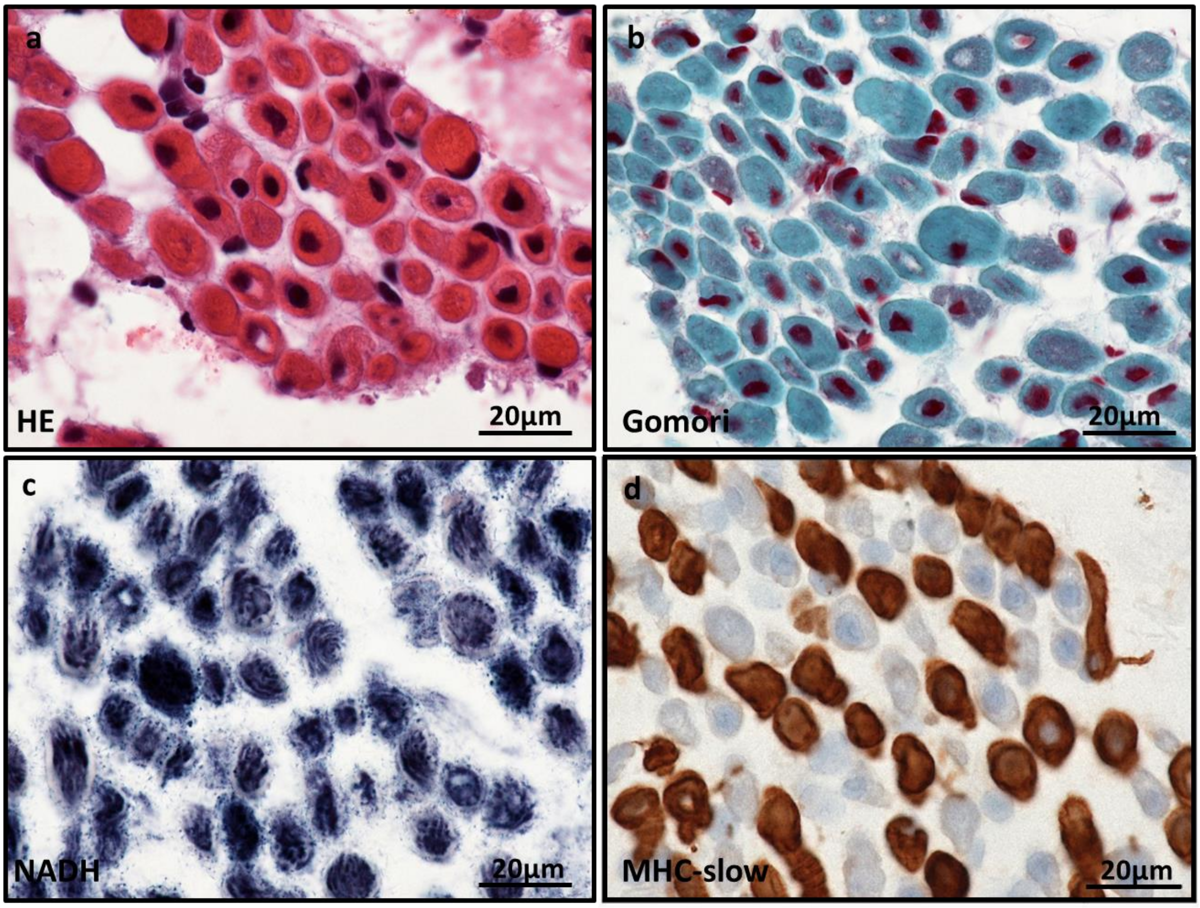

Supplementary Figure 4 Muscle biopsy staining with developmental and fetal myosin and vimentin

Muscle biopsy of patient from this report staining at age of 8 weeks with developmental myosin (MHCd) (a), fetal myosin (MHCn) (b) and Vimentin (c). Staining showed muscle fibers expressing MHCd and in larger amount MHCn and vimentin suggestive for regeneration and delay in skeletal muscle development.

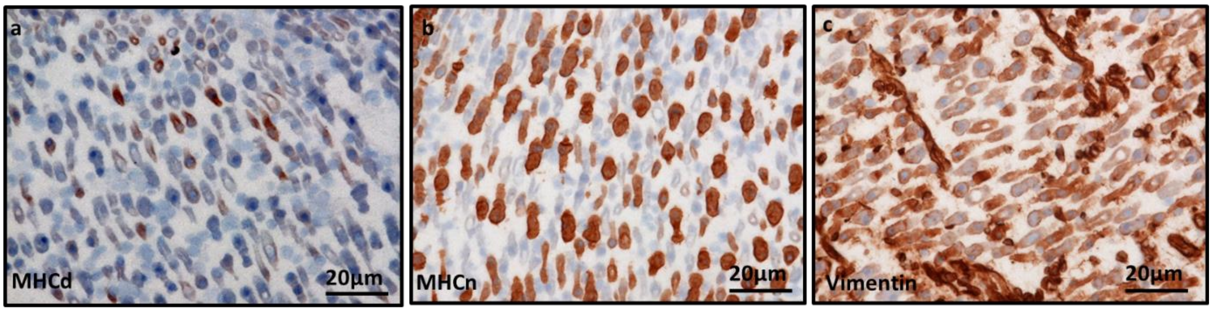

Supplementary Table 1: Rare homozygous variants detected in the patient

Filtered data for rare, homozygous variants showed the genes listed in Supplementary Table 1. After using best practice filtering scheme based on the ACMG guidelines and common prediction tools to see the effect on the proteins by this variants. We analysed the variants by following tools ClinVar (https://www.ncbi.nlm.nih.gov/clinvar/), gnomAD (http://gnomad.broadinstitute.org/), Annovar (http://wannovar.wglab.org) for the prediction of CADD (http://cadd.gs.washington.edu/) and GERP (http://mendel.stanford.edu/SidowLab/downloads/gerp/), SIFT and Polyphen2 (PPH2) predicted by our in-house software Varbank (https://varbank.ccg.uni-koeln.de/), and MutPred (http://mutpred.mutdb.org/), including MutPred-LOF (http://mutpredlof.cs.indiana.edu/) for frameshift and stop-gain variants. The resulting scores for each variant are shown in Supplementary Table 1 and associated data table (Supplementary Table 2) for evaluation of the variants.

On the basis on this analysed data we defined four of the 18 listed variants as possibly pathogenic in following genes: *AIMP2* (Aminoacyl tRNA Synthetase Complex Interacting Multifunctional Protein 2) and *CSAD* (Cysteine sulfinic acid decarboxylase) or as pathogenic to highly pathogenic *OGDH* (2-oxoglutarate dehydrogenase,

mitochondrial) and *SPEG* [8]. After analyzing publications about the genes and the associated phenotypes, we excluded *AIMP2* as patients carrying AIMP2 mutation showed different phenotype such as microcephaly, refractory seizures, intellectual disability, spastic quadriparesis, and do not have a cardiomyopathy [6] and none of these peculiar features were detected in our patient. A disease-modifying effect cannot be excluded but also not proven. Further analysis was done for *CSAD* carrying a splice site variant (Supplementary Table 1). A *Csad* null mouse lacking CSAD indicated that taurine deficiency alters glucose homeostasis and retinal structure [7]. Furthermore, taurine deficiency in *Csad* KO mice let to neonatal lethality (http://www.informatics.jax.org/vocab/mp_ontology/MP:0005386) and *Csad* expression levels were highest during prenatal and early postnatal development [10]. No Mendelian human phenotypes have been associated with CSAD variants to our knowledge and the mice phenotype did not fit with our patient phenotype, besides neonatal lethality. Therefore, we excluded *CSAD* as well. *OGDH* is a catalyzer of a key reaction in the Krebs tricarboxylic acid cycle and we had observed a novel functional variant. However, the clinical phenotype of a classical organic aciduria with hyperlactatemia associated with a congenital deficiency in *OGDH* did not fit to our patients [2]. Thus, it leaves us the *SPEG* mutation as the only pathogenic variant by the ACMG criteria highly consistent with the centronuclear myopathy and even as highly pathogenic by CADD and both parents were heterozygous. The patient has a stop mutation in *SPEG* that is the disease-causing mutation. (NA = Not available, VUS = Variant of Undetermined Significance)

Supplementary Table 1: Rare homozygous variants detected in the patient

**Chr**:position (hg19)	**gene**	**cDNA, protein (RefSeq)**	**ACMG Criteria**	**ACMG**	**ClinVar**	**gnomAD (allele count/allele number)(dbSNP)**	**SIFT**	**PPH**	**CADD**	**GERP**	**MutPred**
**12**:56994738	*BAZ2A*	c.4445G>A, p.R1482Q (NM_013449.3)	PM5, PP2, PP3	VUS (3)	NA	3/245306 (rs759229796)	NA	1	26.30	0.613	0.147
**7**:6049049	*AIMP2*	c.55G>C, p.E19Q (NM_006303.3)	PS4, PM2, PM5, PP2	likely pathogenic (4)	NA	NA	0.1	1	16.87	0.378	0.325
**3**:39307354	*CX3CR1*	c.647A>T, p.Q216L (NM_001337.3)	PM5, PP2	VUS (2)	NA	176/277210 (rs376455816)	NA	1	12.58	0.057	0.131
**7**:63537819	*ZNF727*	c.392A>G, p.N131S (NM_001159522.1)	PM5, PM2	VUS (2)	NA	2/152552	NA	2	2.29	0.165	0.027
**16**:86544885	*FOXF1*	c.710C>T, p.P237L (NM_001451.2)	PM5, PM2	VUS (2)	NA	1/112948	0.1	2	22.50	0.505	0.231
**2:220349304**	***SPEG***	**c.7119C>A, p.Y2373* (NM_005876.4)**	**PVS1, PS3,PM1,PM2, PM4, PP2,PP3**	**pathogenic (7)**	**NA**	**NA**	**NA**	**NA**	**41.00**	**0.329**	**NA***
**21**:34960866	*DONSON*	c.82A>C, p.S28R (NM_017613.2)	PM2, PM5	VUS (2)	Conflicting interpretations of pathogenicity	32828 (rs768071555)	0.1	1	14.32	0.217	0.123
**3**:52090300	*DUSP7*	c.83G>A, p.G28E (NM_001947.3)	PM2, PP2	VUS (2)	NA	1/28972	0.1	NA	5.73	0.164	0.109
**12**:58126199	*AGAP2*	c.1781C>A, p.P594Q (NM_001122772.2)	PM2, PP3	VUS (2)	NA	19/258592 (rs138992134)	NA	**3**	**29.60**	0.478	0.247
**22**:50278100	*ZBED4*	c.790G>A, p.D264N (NM_014838.2)	PM2	VUS (1)	NA	25/246158 (rs748633146)	NA	2	14.92	0.519	0.129
**7**:44746938	*OGDH*	c.2747G>T, p.R916L (NM_002541.3)	PS3, PM2, PP3, PP2	likely pathogenic (4)	NA	NA	NA	**3**	**34.00**	**0.864**	**0.927**
**2**:219603001	*TTLL4*	c.602T>C, p.I201T (NM_014640.4)	PM2, PP2	VUS (2)	NA	NA	NA	2	21.30	0.544	0.084
**2**:231973829	*HTR2B*	c.848T>C, p.M283T (NM_000867.4)	PM5, PM2, PP3	VUS (3)	NA	14/246030 (rs370382293)	**0.8**	2	11.58	**0.921**	0.148
**21**:45959933	*KRTAP10-1*	c.101T>C, p.L34P (NM_198691.2)	PM5, PM2	VUS (2)	NA	79/276716 (rs200714107)	1	1	0.002	0.217	0.082

**16**:89985770	*MC1R*	c.104G>A, p.C35Y (NM_002386.3)	PM5, PM2, PP3	VUS (3)	Uncertain significance	54/275948 (rs758135673)	NA	**3**	**23.00**	**0.555**	**0.745**
**12**:53554105	*CSAD*	c.526-2A>G, NM_001244706.1	PS3, PM1, PM2, PP3	likely pathigenic (4)	NA	NA	NA	NA	**23.66**	0.37	NA*
**21:**34861336_34861341del TCTTCT	*DNAJC28*	c.360_365delAGAAGA, p.E120_E121del (NM_017833.3)	PM2, PM4, PP3	VUS (3)	NA	NA	NA	NA	15.77	NA	NA*
**21**:132313098_132313099insGCTGCCGCT	*MMP17*	c.59_60insGCTGCCGCT, p.L23_P24insLPL (NM_016155.4)	PM2, PM4, PP3	VUS (3)	NA	NA	NA	NA	NA	NA	NA*

* Analyzed by MutPred-LOF, one software of MutPred for frameshift and stop-gain variants (http://mutpredlof.cs.indiana.edu/)

Supplementary Table 2: Scores of prediction tools

Tool	highly pathogenic	pathogenic/probably damaging	likely pathogenic/possible damaging	likely benign/benign	benign/ unknown
SIFT		>0,5			
PPH2		3	2	1	0
CADD	>30	>20	15-20	<15	<10
GERD		>0,5			
Mutpred		>0,5			
ACMG [5]		1 very strong (PVS1) and 1 strong (PS1-PS4) or 2 strong or 1 strong and 3 moderate (PM1-PM6)	1 very strong and 1 moderate or 1 strong and 1 moderate or 1 strong and 2 supporting (PP1-PP5)	1 strong (BSA-BS4) and 1 supporting (BP1-BP7) or 2 supporting	1 stand-alone (BA1) or 2 strong

Reference:

1. Blum AS, Rutkove SB. (2007) The Clinical Neurophysiology Primer. Springer, Berlin.
2. Guffon N, Lopez-Mediavilla C, Dumoullin R, et al. (1993) 2-Ketoglutarate dehydrogenase deficiency, a rare cause of primary hyperlactatemia: report of a new case. J Inherit Metab Dis. 16(5): p. 821-30.
3. Gutrecht JA, Dyck PJ (1970) Quantitative teased-fiber and histologic studies of the human sural nerve during postnatal development. The Journal of comparative neurology. 138: 117-129.
4. Jacobs JM, Love S (1985) Qualitative and quantitative morphology of human sural nerve at different ages. Brain. 108 (Pt 4): 897-924.
5. Richards S, Aziz N, Bale S et al (2015) Standards and guidelines for the interpretation of sequence variants: a joint consensus recommendation of the American College of Medical Genetics and Genomics and the Association for Molecular Pathology. Genet Med. 17(5): p. 405-24.
6. Shukla A, Das Bhowmik A, Hebbar M, et al. (2018) Homozygosity for a nonsense variant in AIMP2 is associated with a progressive neurodevelopmental disorder with microcephaly, seizures, and spastic quadriparesis. J Hum Genet. 63(1): p. 19-25.
7. Sidime F, Phillips G, LaMassa N et al. (2017) Glucose Homeostasis and Retinal Histopathology in CSAD KO Mice. Adv Exp Med Biol. 975: p. 503-511.
8. Wang H, Castiglioni C, Kacar Bayram A et al. (2017) Insights from genotype-phenotype correlations by novel SPEG mutations causing centronuclear myopathy. Neuromuscul Disord. 27(9):836-842.
9. Weis J, Brandner S, Lammens M, et al. (2012) Processing of nerve biopsies: a practical guide for neuropathologists. Clin Neuropathol. 31(1):7-23.
10. Winge, I., Teigen K, Fossbakk A et al. (2015) Mammalian CSAD and GADL1 have distinct biochemical properties and patterns of brain expression. Neurochem Int. 90: p. 173-84.
